# Data set for diet specific differential gene expression analysis in three *Spodoptera* moths

**DOI:** 10.1016/j.dib.2016.04.029

**Published:** 2016-05-20

**Authors:** A. Roy, W.B. Walker, H. Vogel, S.K. Kushwaha, S. Chattington, M.C. Larsson, P. Anderson, D.G. Heckel, F. Schlyter

**Affiliations:** a Chemical Ecology, Department of Plant Protection Biology, Swedish University of Agricultural Sciences, SE-230 53 Alnarp, Sweden; b Department of Plant Breeding, Swedish University of Agricultural Sciences, SE-230 53 Alnarp, Sweden; c Department of Evolutionary Neuroethology, Max Planck Institute for Chemical Ecology, Hans-Knoell-Strasse 8, 07745 Jena, Germany; d Department of Entomology, Max Planck Institute for Chemical Ecology, Hans-Knoell-Strasse 8, 07745 Jena, Germany

**Keywords:** Differential expression analysis (DGE), Transcriptomics, *Spodoptera*, Adaptation, Generalist, Specialist, RPKM (reads per kilo base of transcript per million mapped reads), RNA seq

## Abstract

Examination of closely related species pairs is suggested for evolutionary comparisons of different degrees of polyphagy, which we did here with three taxa of lepidopteran herbivores, *Spodoptera* spp (*S. littoralis*, *S. frugiperda* maize (C) and rice (R) strains) for a RNAseq analysis of the midguts from the 3rd instar insect larvae for differential metabolic responses after feeding on pinto bean based artificial diet vs maize leaves. Paired-end (2×100 bp) Illumina HiSeq2500 sequencing resulted in a total of 24, 23, 24, and 21 million reads for the SF-C-Maize, SF-C-Pinto, SF-R-Maize, SF-R Pinto, and a total of 35 and 36 million reads for the SL-Maize and SL-Pinto samples, respectively. After quality control measures, a total of 62.2 million reads from SL and 71.7 million reads from SF were used for transcriptome assembly (TA). The resulting final *de novo* reference TA (backbone) for the SF taxa contained 37,985 contigs with a N50 contig size of 1030 bp and a maximum contig length of 17,093 bp, while for SL, 28,329 contigs were generated with a N50 contig size of 1980 bp and a maximum contig length of 18,267 bp. The data presented herein contains supporting information related to our research article Roy et al. (2016) http://dx.doi.org/10.1016/j.ibmb.2016.02.006[1].

## Graphical abstract

**Figure:**
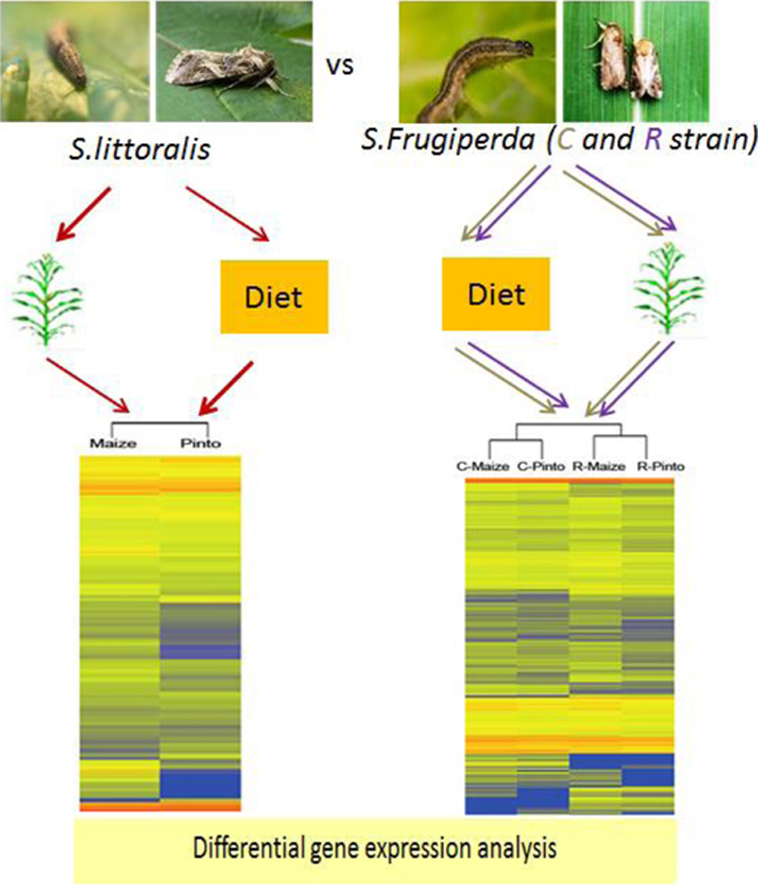

**Specifications Table**

**Table:** 

Subject area	Biology
More specific subject area	Molecular Ecology and Bioinformatics
Type of data	Tables, figures, charts, and Excel files
How data was acquired	High-throughput RNA-sequencing using Illumina HiSeq2500 instrument at the Max Planck Genome Centre Cologne (MPGCC).
Data format	Analyzed
Experimental factors	RNA Isolation, cDNA library construction, and sequencing
Experimental features	Differential midgut gene expression analysis of three closely related *Spodoptera* moths feeding on pinto diet vs maize leaves.
Data source location	Alnarp, Sweden and Jena, Germany
Data accessibility	Data is available with this article. Raw data is publically deposited with the following accession number: PRJEB 19473 [EBI short read archive (SRA)], SRA Ids - ERR986577 to ERR986580.

**Value of the data**

• The genetic information of *Spodoptera* taxa in public databases is scant.
• The gene expression data provide insights into the adaptive mechanisms of these closely *Spodoptera* with relatively little phylogenetic noise [2].
• It is a significant contribution to further research on adaptive mechanisms in *Spodoptera* taxa at all levels.
• The data could be used as a benchmark and for comparative or collaborative studies in other noctuid moths (Nocutidae, the largest family in the Lepidoptera).
• This data further supports our publication [1] and the use of transcriptomic technologies in non-model organisms.

## 1 Data

We provide data on the transcriptome responses in the larval guts of the three moth taxa when feeding on a semi-artificial diet (suitable for all three insect groups) and on maize leaves (the primary food of only the *S. frugiperda* C (Corn) strain). The raw data of our study on *Spodoptera* larval responses to different diets [1], can be accessed directly at the following URL: http://www.ebi.ac.uk/ena/data/view/PRJEB10473.

**Fig. 1:**
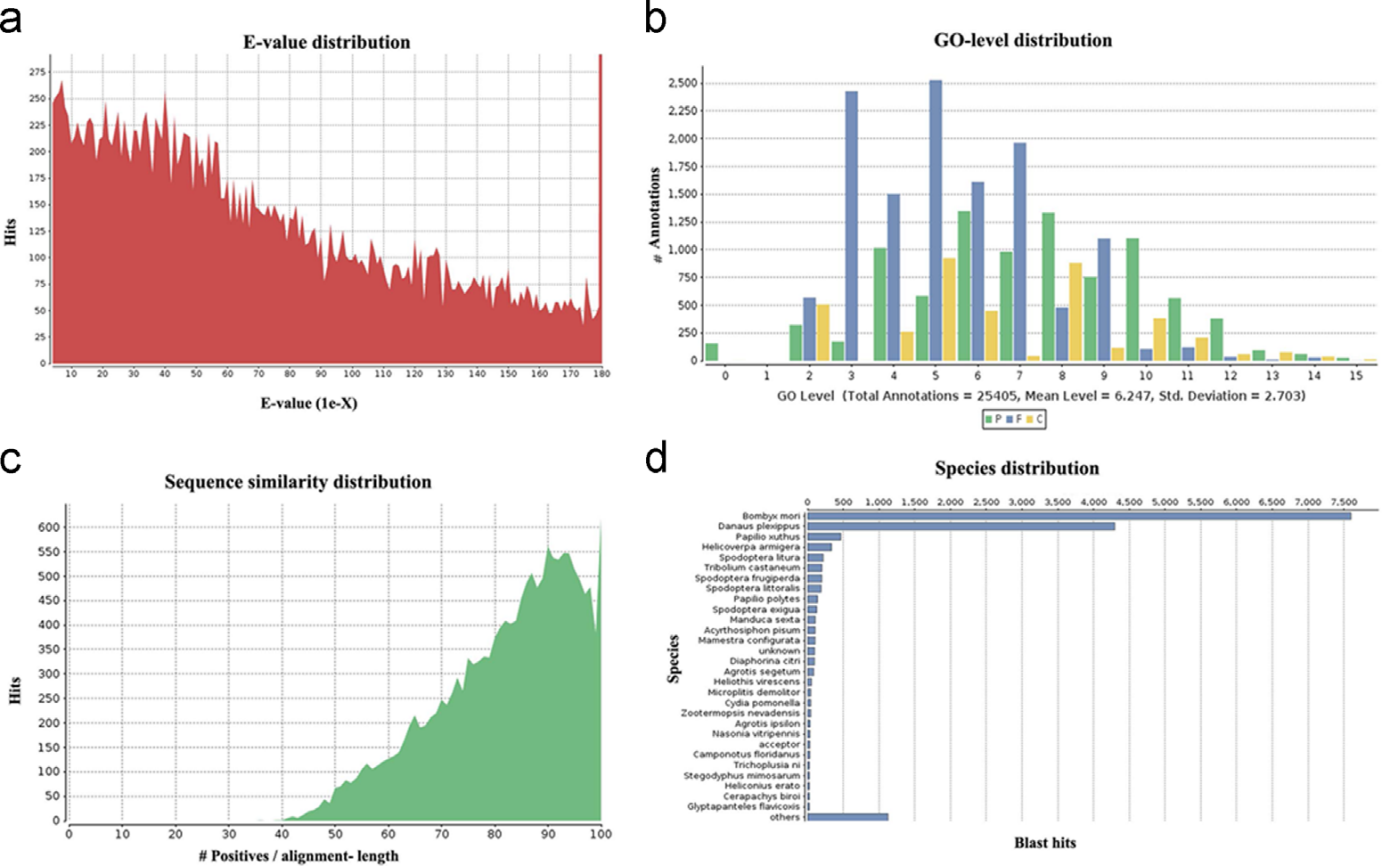
Homology analysis output of SL unigenes. a) E-value distribution for top BLAST hit for unique sequence, b) GO level annotation distribution of the top BLAST hits, c) similarity distribution for top BLAST hit for unique sequence, d) species distribution for top BLAST hit for each unique sequence.

**Fig. 2:**
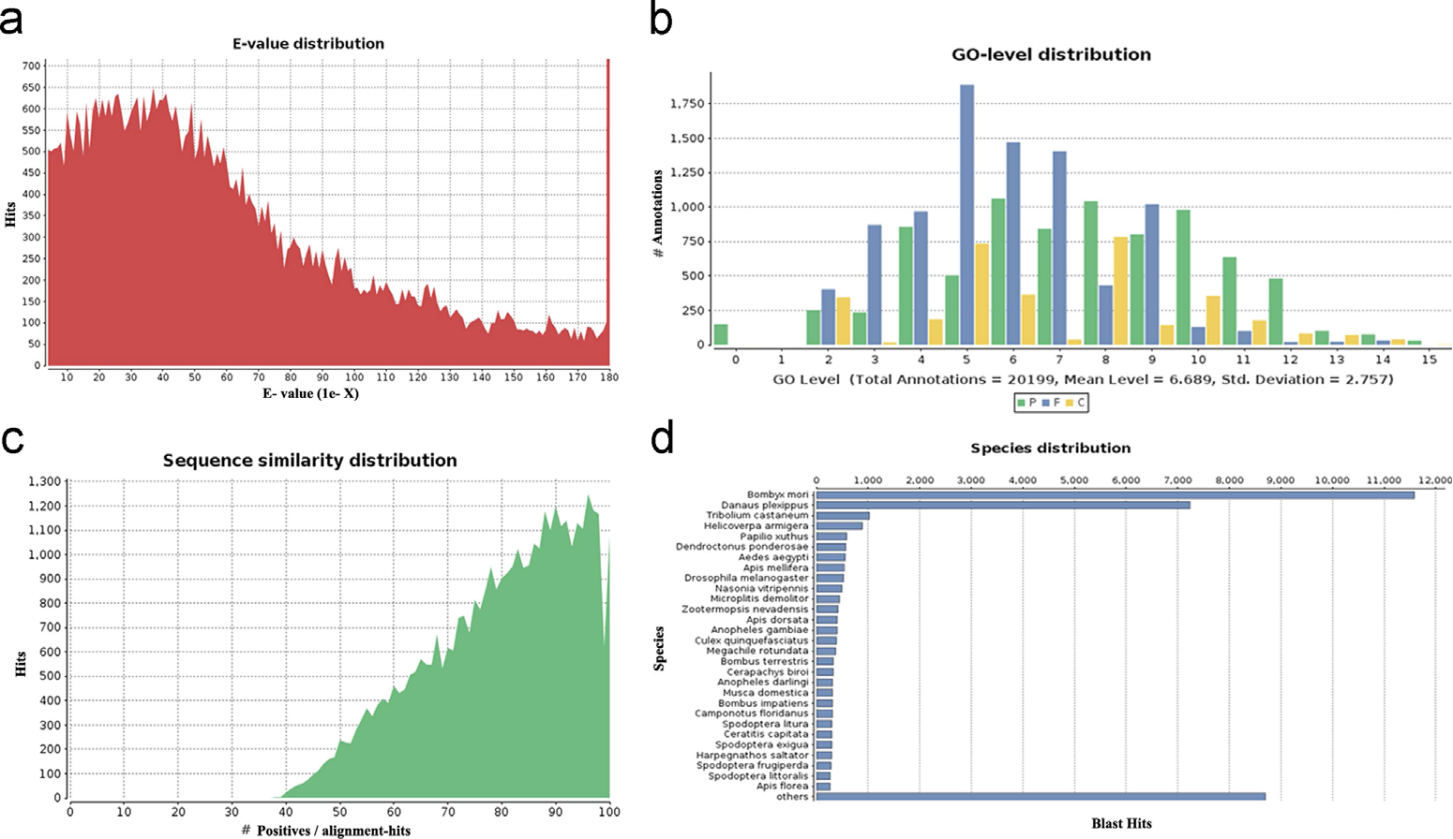
Homology analysis output of SF unigenes. a) E-value distribution for top BLAST hit for unique sequence, b) GO level annotation distribution of the top BLAST hits, c) similarity distribution for top BLAST hit for unique sequence, d) species distribution for top BLAST hit for each unique sequence.

**Fig. 3:**
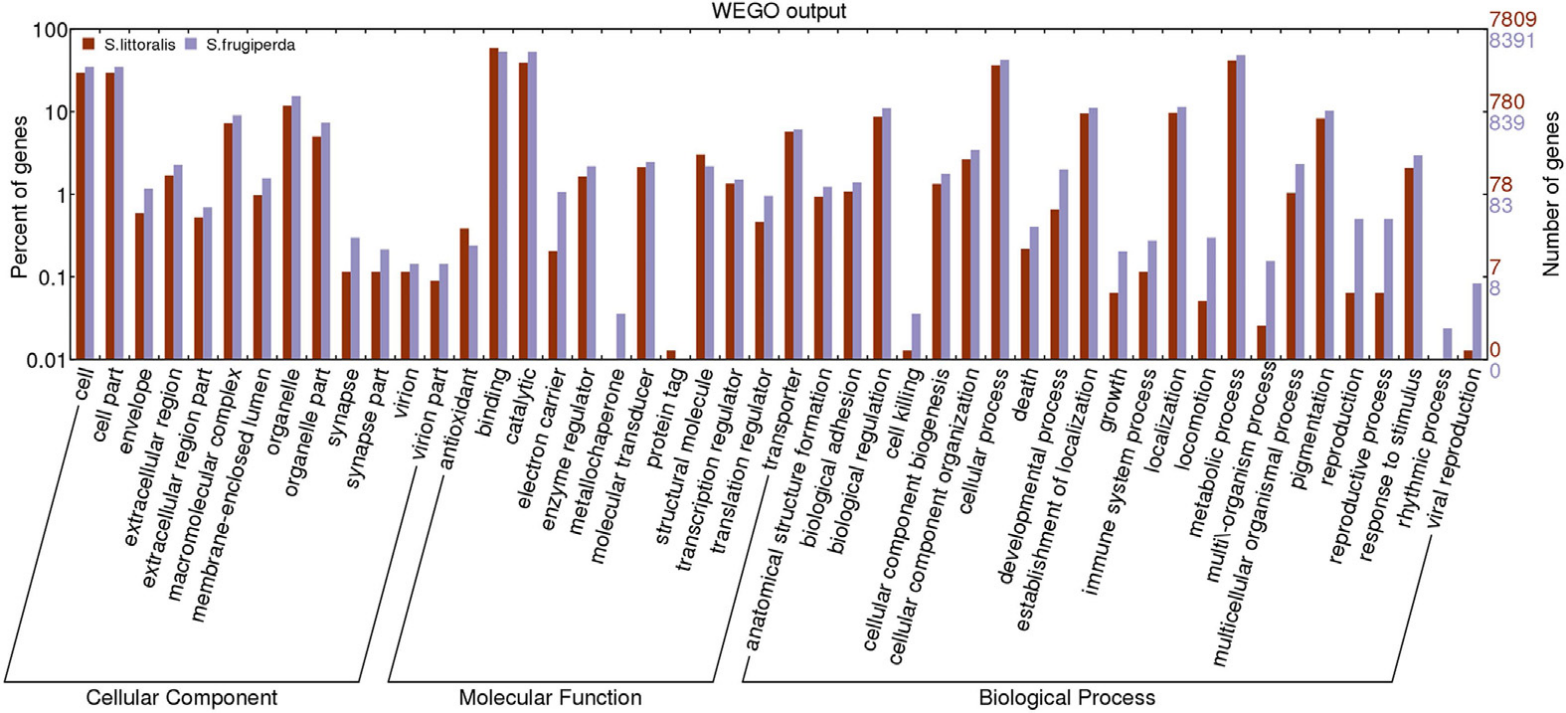
WEGO plot showing gene ontology classification of SL and SF unigene sets. Classification and functional distribution of the 7809 unigenes of the SL and 8391 unigenes of the SF transcriptome according to the 3 major classifications of gene ontology: Biological Process, Molecular Function and Cellular Component.

**Fig. 4:**
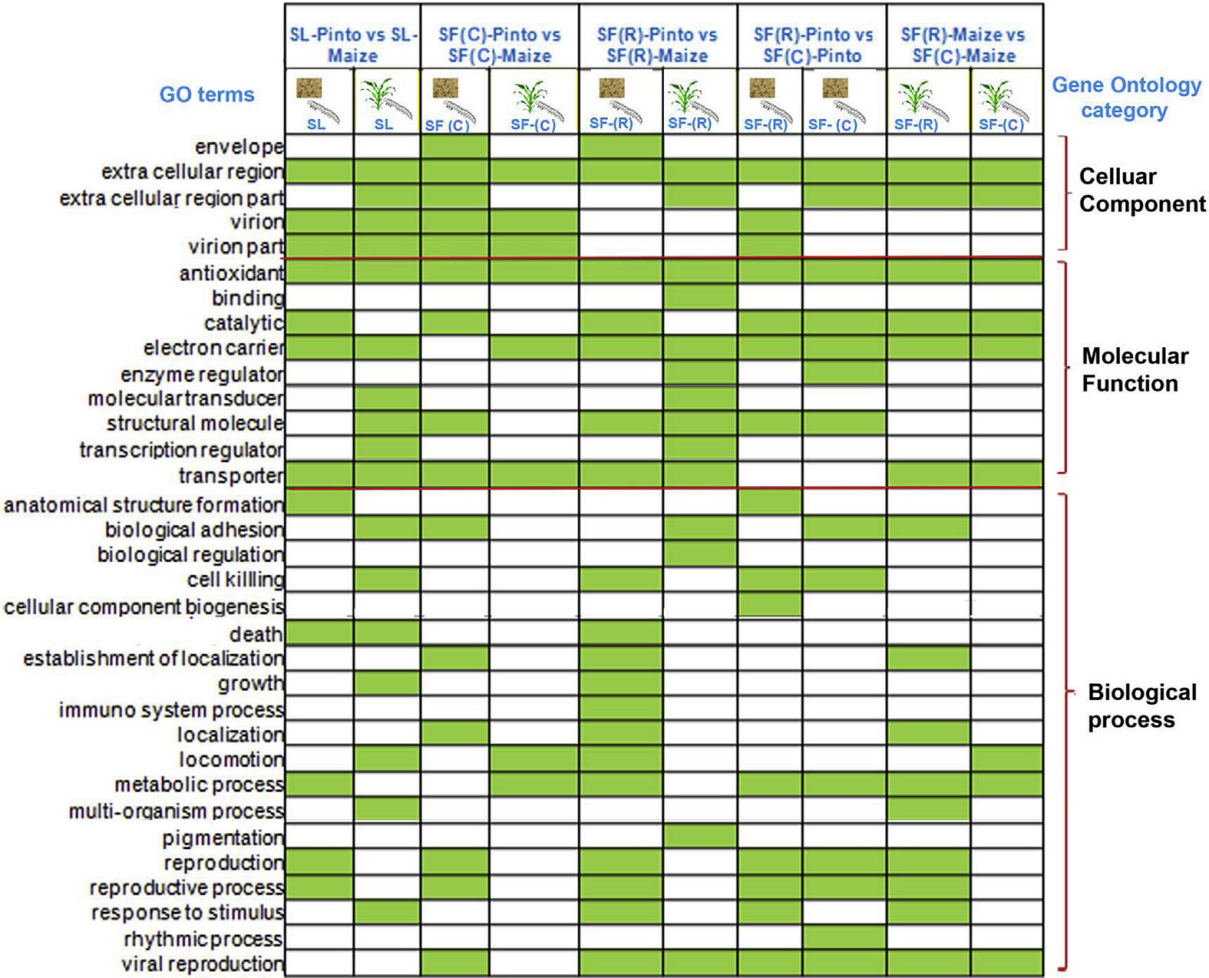
Summary of GO enrichment analysis. Green boxes show enrichment events for the corresponding GO terms.

**Fig. 5:**
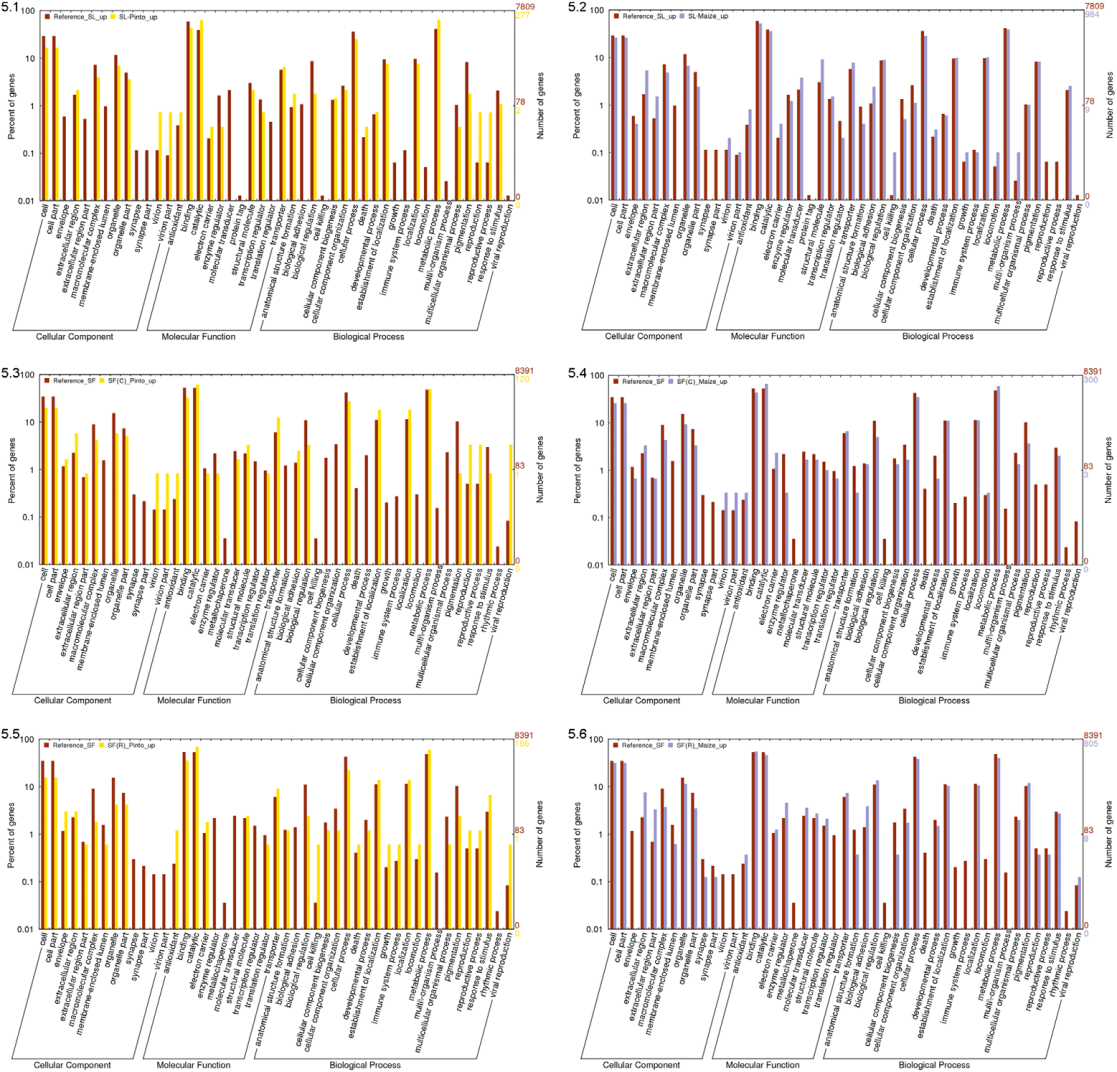

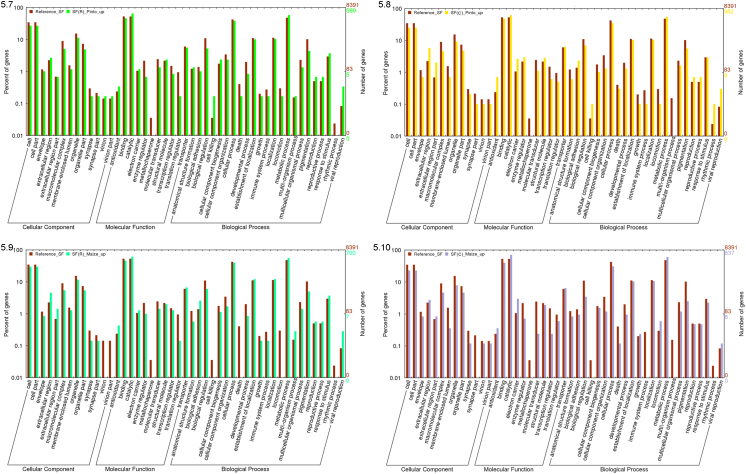
WEGO output showing over-represented GO terms against reference transcriptome for each species after feeding on pinto bean diet (P) and maize leaves (M). 5.1 & 5.2) SL-P vs SL-M, 5.3 & 5.4) SF-C-P vs SF-C-M , 5.5 & 5.6) SF-R-P vs SF-R-M, 5.7 & 5.8) SF-C-P vs SF-R-P, and 5.9 & 5.10) SF-C-M vs SF-R-M.

The analyzed data presented here contains three types of data 1) the transcriptomes of *S. littoralis* and *S. frugiperda* 3rd instar larval midgut (assembled and annotated) in *two Excel files (plain text & numerical)* (Supplemental Files 1 and 2), 2) Quality control and annotation plots as Blast2GO, WEGO (Web Gene Ontology Annotation) in *five Figures (plain graphical)* (Fig. 1, Fig. 2, Fig. 3, Fig. 4, Fig. 5), and 3) differential gene expression (DGE) analysis in *two sets of Supplementary charts (graphical listings of numbers)* (Supplementary charts 6.1-6.5 and 7.1-7.6).

## 2 Experimental design, materials and methods

### 2.1 Plant and insect

The detailed information for plants and insect strains (incl. origin) will be found in our publication associated with this data set. In brief, maize plants (Sweet Nugget F1) were grown in Alnarp at 23 °C and 70% RH. The R strain of *S. frugiperda* (Moore Haven, Florida), C strain of *S. frugiperda* (Santa Isabel, Puerto Rico) and *S. littoralis* (Egypt) were maintained in the lab on pinto bean based semi-artificial diet for several generations prior to the experiments.

### 2.2 RNA isolation

Total RNA was extracted from each of the larval midgut samples using a combined approach of Trizol based extraction followed by purification using the RNeasy Mini Kit (Qiagen, Venlo, Netherlands) following the manufacturer׳s guidelines. DNA was eliminated with on-column RNase-free DNase (Qiagen). The integrity of the RNA was verified using an Agilent 2100 Bioanalyzer and a RNA 6000 Nano Kit (Agilent Technologies, Palo Alto, CA). The quantity of RNA was determined using a Nanodrop ND-1000 spectrophotometer. RNA from a total of twenty individuals was combined in one pool per sample (for each of the SF-C-Maize, SF-C-Pinto, SF-R-Maize, SF-R-Pinto and for the SL-Maize and SL-Pinto samples) for sequencing.

### 2.3 RNA seq data generation and assembly

Tissue-specific transcriptome sequencing of four different RNA samples for SF and two RNA samples for SL was carried out on an Illumina HiSeq2500 Genome Analyzer platform using paired-end (2×100 bp) read technology with RNA fragmented to an average of 150 nucleotides at the Max Planck Genome Centre Cologne (MPGCC). Sequencing resulted in a total of 24, 23, 24, and 21 million reads for the SF-C-Maize, SF-C-Pinto, SF-R-Maize, SF-R Pinto, and a total of 35 and 36 million reads for the SL-Maize and SL-Pinto samples, respectively. Quality control (QC) measures which include the filtering of high-quality reads based on the quality score given in fastq files, removal of reads containing primer/adaptor sequences and trimming of read length, were performed using CLC Genomics Workbench v6.5 (http://www.clcbio.com). The *de novo* transcriptome assembly was then carried out using the Genomics Workbench v6.5 software and the selection of the presumed optimal consensus transcriptome was done using the pre-optimized approach [3]. Any conflicts among the individual bases were resolved by selecting the base with highest frequency. Contigs shorter than 250 bp were removed from the final analysis.

The *de novo* assembled transcriptomes were annotated using BLAST, Gene Ontology and InterProScan searches using BLAST2GO PRO v2.8.1 (www.blast2go.de) [4]. For BLASTX searches [5] against the non-redundant NCBI protein database (NR database) up to the 20 best NR hits per transcript were retained, with an E-value cut-off≤10^−1^ and a minimum match length of 15 amino acids to obtain the best homologue for the predicted short polypeptides. Annex [6] was used to optimize the GO term identification further by crossing the three GO categories (biological process, molecular function and cellular component) to search for name similarities, GO term relationships and enzyme relationships within metabolic pathways (Kyoto Encyclopedia of Genes and Genomes) [7], [8].

Finally, the GO enrichment analysis (level 2) was performed by plotting all the GO information, but from only the contigs with an expression difference more than fourfold and a minimum RPKM cut off at 1 against the reference set (SL and SF whole transcriptome) using the web tool called WEGO [9].

### 2.4 Re-mapping and digital gene expression analysis

Digital gene expression analysis was performed by using QSeq Software (DNAStar Inc.) to re-map the Illumina reads from all individual samples onto their respective transcriptome backbone reference, and then count the sequences to estimate expression levels, using previously described parameters for read mapping and normalization such as *n*-mer length=25; read assignment quality options required at least 25 bases and at least 90% of bases matching within each read to be assigned to a specific contig; maximum number of hits for a read=10; *n*-mer repeat settings were automatically determined by the software and other default settings [3]. Biases in the sequence datasets and different transcript sizes were corrected using the RPKM algorithm (reads per kilobase of transcript per million mapped reads; log2 transformed) to obtain accurate estimates for relative expression levels. To control for the effect of global normalization using the RPKM method, we also analyzed a number of highly-conserved housekeeping genes that are frequently used as control genes for quantitative RT-PCR. These included several genes encoding ribosomal proteins (RpL5, RpL8, RpL7a, RpL15, RpL22, RpS3a, RpS5, RpS8, RpS15, RpS18, and RpS20), elongation factor 1-alpha and eukaryotic translation initiation factors 4b and 5a. The corresponding genes were evaluated for overall expression levels across samples and treatments and were found to display expression level differences (based on log2-transformed RPKM values) lower than 1.4-fold across samples, validating them as housekeeping genes with no diet or strain dependent differential expression. Due to the robust pooling strategy detailed above, combining large numbers (20) of larvae per sample and treatment group, our effective sample size per group is *n*=1. Hence, there is no statistical power for conventional hypothesis testing using this pooling approach. However, this pooling strategy reduces the influence of individual outliers. Expression differences in the gene categories with less than 10 total identified transcripts were not considered for analysis (Supplementary charts 6.1-6.5).

## Appendix A Supplementary material

Supplementary material: **File 1.** RNA-Seq assembly, contig sequences, Blast2GO hits against NR database, hit accessions, GO annotations and relative expression levels for *Spodoptera littoralis* (Excel file: Gut_transcriptome_S-littoralis_Supplementary File 2.xlsx). **File 2.** RNA-Seq assembly, contig sequences, Blast2GO hits against NR database, hit accessions, GO annotations and relative expression levels for *Spodoptera frugiperda* (Excel file: Gut_transcriptome_S-frugiperda_Supplementary File 2.xlsx). **Supplementary charts 6.1–6.5.** Detailed expression profile of transcripts associated with key midgut functions like digestion, detoxification, immunity, transport, signalling, growth, peritrophic matrix, etc. in SL, SF-C and SF-R after feeding on pinto bean diet or maize leaves. **Supplementary charts 7.1–7.6.** List showing **e**xpression distribution of top 50 over- expressed and under-expressed transcripts in *Spodoptera* taxa (SL, SF-C, and SF-R) after feeding on maize leaves, relative to feeding on the artificial pinto diet.

## Footnotes

Appendix A Supplementary data associated with this article can be found in the online version at doi:10.1016/j.dib.2016.04.029.
