# Supplementary material for: Data set for diet specific differential gene expression analysis in three Spodoptera moths
Source: Data Brief. 2016 May 20;8:448–55. doi: 10.1016/j.dib.2016.04.029 (PMC4910185; doi:10.1016/j.dib.2016.04.029)
Supplement: Supplementary file 1 — Supplementary material: File 1. RNA-Seq assembly, contig sequences, Blast2GO hits against NR database, hit accessions, GO annotations and relative expression levels for Spodoptera littoralis (Excel file: Gut_transcriptome_S-littoralis_Supplementary File 2.xlsx). File 2. RNA-Seq assembly, contig sequences, Blast2GO hits against NR database, hit accessions, GO annotations and relative expression levels for Spodoptera frugiperda (Excel file: Gut_transcriptome_S-frugiperda_Supplementary File 2.xlsx). Supplementary charts 6.1–6.5. Detailed expression profile of transcripts associated with key midgut functions like digestion, detoxification, immunity, transport, signalling, growth, peritrophic matrix, etc. in SL, SF-C and SF-R after feeding on pinto bean diet or maize leaves. Supplementary charts 7.1–7.6. List showing expression distribution of top 50 over- expressed and under-expressed transcripts in Spodoptera taxa (SL, SF-C, and SF-R) after feeding on maize leaves, relative to feeding on the artificial pinto diet. [file mmc1.zip › DIB_Supplementary Charts 7.1.doc]

**Supplementary Charts 7**

7.1 SL-Maize (Up 50)

7.2 SL-Maize (Down 50)

7.3 SF-R-Maize (Up 50)

7.4 SF-R-Maize (Down 50)

7.5 SF-C-Maize (Up 50)

7.6 SF-C-Maize (Down 50)
